# Efficiency of octenidine dihydrochloride alcohol combination compared to ethanol based skin antiseptics for preoperative skin preparation in dogs

**DOI:** 10.1371/journal.pone.0293211

**Published:** 2023-11-07

**Authors:** Fabian Eigner, Stefanie Keller, Sarah Schmitt, Sabrina Corti, Mirja C. Nolff

**Affiliations:** 1 Vetsuisse Faculty Zürich, Clinic for Small Animal Surgery, University of Zürich, Zurich, Switzerland; 2 Vetsuisse Faculty Zürich, Clinic for Small Animal Reproduction, University of Zürich, Zurich, Switzerland; 3 Vetsuisse Faculty Zürich, Section of Veterinary Bacteriology, Institute for Food Safety and Hygiene, University Zürich, Zurich, Switzerland; 4 Vetsuisse Faculty Zürich, Institute for Food Safety and Hygiene, University Zürich, Zurich, Switzerland; Universidad Autonoma de Chihuahua, MEXICO

## Abstract

**Objective:**

To quantify the bacterial burden after skin disinfection using an alcohol octenidine dihydrochloride combination (Octenisept®) compared to an 74.1% ethanol 10% 2-propanol combination (Softasept N®).

**Study design:**

Prospective randomized clinical trial.

**Material & methods:**

61 dogs undergoing clean or clean-contaminated surgeries (excluding surgeries on the gastrointestinal tract) were randomly assigned to group O (skin disinfection with alcohol and octenidine dihydrochloride after washing with octenidine containing soap) or to control group C (skin disinfection using the ethanol-2-propanol combination after washing with a neutral soap without antiseptic ingredients). Samples were then taken from 8 different locations within the surgical field at four different stages: after clipping, after washing, after disinfection and one hour later. At each stage, two different sampling techniques (wet-dry swab technique (WDS) and contact plates (CP)) were used for quantitative analysis of bacterial counts.

**Results:**

WDS detected about 100-fold more bacteria compared to CP sampling in cases with high bacterial burden, but was not accurate enough to detect small numbers. CP sampling was therefore used for comparison of treatment protocols. 30 dogs were assigned to group O and 31 to group C. A relative reduction of 69% in group O and 77 percent in group C was observed after the soap wash. No significant differences were detected between both groups. Washing and disinfection resulted in a reduction of bacterial counts of 99.99% in group O versus 99.7% in group C (p = 0.018). Bacterial reduction one hour after washing and disinfection was significantly higher in group O (99.9%) than in group C (98.5%, p = 0.001).

**Conclusion:**

Additional octenidine dihydrochloride provided a slightly better decontamination effect after disinfection, particularly one hour after, which means it may only be indicated in longer surgeries. WDS is more sensitive but less specific to detect bacteria on the skin than the CP sampling.

## Introduction

Surgical site infections (SSIs) are the second most common cause of hospital acquired infections in veterinary and human medicine [1–3]. SSIs are a burden for the patient and the owner, they cause an increase of health care costs and of antibiotics use and can be associated with development of multidrug resistant bacteria [4,5]. Due to the considerable threat of resistance formation, the European Parliament in summer 2021 restricted antibiotic use in veterinary medicine considerably [6]. In order to decrease the overall antibiotic selection pressure, prudent use is a mandatory requirement [7].

To prevent SSIs, several preventive measures were established. Optimized perisurgical and surgical management remains an important factor in this context, such as atraumatic surgical techniques, avoiding hypotension, shortening anesthesia time, shortening surgical time, aseptic use of propofol, decreasing number of persons in the operating room, clipping within 4 hours before surgery, maintaining the skin barrier intact and optimized scrubbing and disinfection [8–16]. It has been shown in humans that optimized perioperative hygiene can help to reduce perioperative prophylactic antibiotic administration, while simultaneously maintaining stable SSI rates in many procedures [7].

Recent guidelines for clean and clean-contaminated procedures in small animals clearly advocate prudent use of perioperative antibiotics [17–20], consequently the perioperative hygiene is more important than ever. Perioperative skin disinfection is a crucial point [21], because 80% of bacteria causing SSIs originate from the patient’s skin [2]. Despite this, there have only been a few published studies about skin antisepsis in dogs [22–34]. Chlorhexidine remains the most frequently used antiseptic in veterinary and human medicine, even though not enough studies are available proving a clear benefit of using chlorhexidine containing antiseptics when compared to other alcohol based skin preparations without additional biocide [4,22,23,26,27,29]. This recommendation for skin antisepsis is also contradictory to current guidelines of wound antisepsis which state that chlorhexidine should be replaced by modern antiseptics [35–37]. The reasons being the progressive development of resistant microbes (both against antiseptic and antibiotics), and the unfavorable biocompatibility index of chlorhexidine compared to modern antiseptics, such as octenidine [3,35,38–46].

Octenidine is a modern biocide and represents an alternative to already longer established substances such as chlorhexidine or polyvidone-iodine [47]. It has a broad antimicrobial spectrum and is about 3–10 times more efficient than chlorhexidine *in vitro* [47]. For skin antisepsis prior to surgical procedures, fixed combinations of octenidine with aliphatic alcohols are available [47,48]. The idea behind these combinations is to add a sustained antimicrobial effect (residual effect) to the immediate antimicrobial action of alcohols [47,49].

Octenidine dihydrochloride in combination with aliphatic alcohols have been licensed for perioperative skin antisepsis. However, there is no published research showing clear benefits of using a biocide on top of alcohol-based products, or any that investigates octenidine [21,28]. Moreover, biocides have been linked to resistance and cross-resistance to antibiotics, therefore it seems crucial to prove an additional effect prior to broad usage [3,50,51]. Further, certain side effect of skin antisepsis including blistering, necrosis and scarring have been described for newborns after the use of octenidine. For adults, severe side effects like the ones described in newborns are not known, but contact dermatitis and tissue reactions such as swelling have been reported [52,53]. Irrigation of deep wounds, particularly bite wounds, using octenidine dihydrochloride without drainage may lead to persistent edematous changes, inflammatory reactions and necrosis in dogs [54]. Overall octenidine dihydrochloride has a high biocompatibility index compared to other biocides [38,40,55].

Alcohols such as 70–80% ethanol kill vegetative bacteria within 10–90 seconds in suspension tests [2]. In addition, they are active against *Mycobacterium* spp., fungi, and viruses but have no activity against bacterial spores [56]. Important: Optimal bactericidal activity is achieved at a concentration of 60% to 90% [57]. Alcohols like ethanol and isopropanol are only slightly toxic to the skin, not allergenic and do not stain tissue [2].

The overall aim of this prospective, randomized, and partially blind study was to validate the postulated benefit of additional octenidine dihydrochloride compared to alcohol only for perioperative skin antisepsis.

The primary objective of this study was to quantify the bacterial load after skin disinfection with alcohol plus octenidine dihydrochloride compared to alcohol only. We hypothesized that the ethanol skin antisepsis would be equivalent to octenidine dihydrochloride alcohol with regard to bioburden reduction.

The secondary objective of this study was to compare two sampling techniques for quantitative analysis of bacteria on the skin: the wet-dry swab (WDS) versus the contact plate (CP). We hypothesized that the WDS technique is more sensitive than the CP for quantifying the number of bacteria on the skin.

## Materials and methods

### Inclusion criteria

The study protocol was accepted by the legal authorities under the license number ZH 178/19. Dogs were enrolled if their body weight was above 5 kg, considered ASA I or II (21), and had clean or clean-contaminated surgeries (not affecting the GI tract) that were performed without perioperative antibiotic prophylaxis. Included procedures were ovariectomy, ovariohysterectomy, laparoscopic ovariectomy, splenectomy, cystotomy, skin tumor excision, nephrectomy, lymphadenectomy, adrenalectomy, amputation of limb and digit, esophagus myotomy, fixation of a chronic diaphragmatic hernia or of an ectopic ureter. The draped field of skin had to be big enough to allow consecutive sampling of separate skin areas using both techniques without overlapping the sampled zones. Finally, owners had to give written consent to enroll their dogs in the study.

### Exclusion criteria

Dogs were excluded if the surgery required perioperative antibiotic coverage, the clipped skin area was smaller than 900 cm^2^, dogs had an ASA score of III or more, the procedure was contaminated or infected, if inadvertent contamination of the surgical field occurred during a clean or clean contaminated procedure or if the owners declined consent.

### Sampling technique

**Fig 1:**
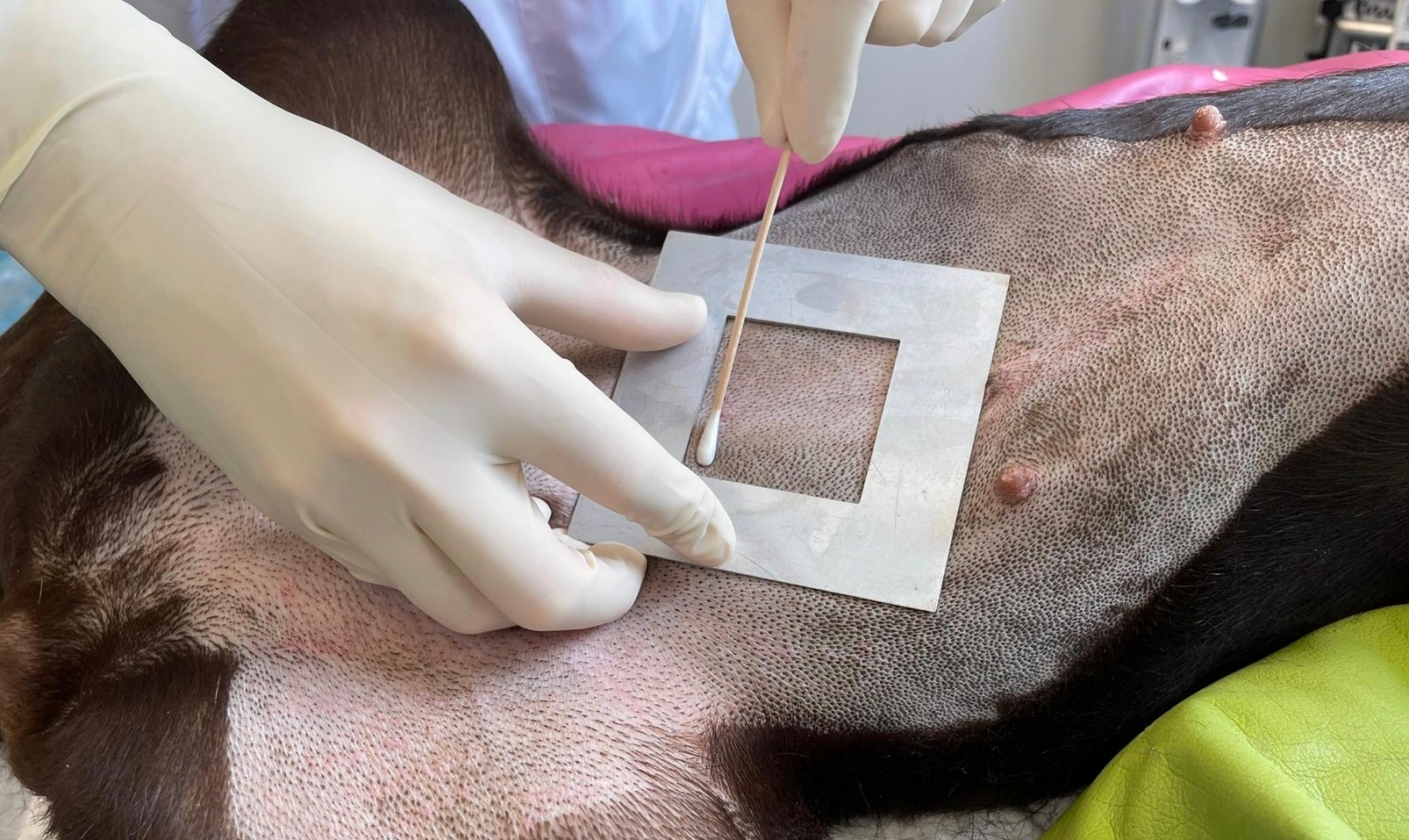
Wet-dry swab (WDS) technique on the skin of a Labrador Retriever. Printed under a CC BY license, with permission from Fabian Eigner, original copyright [2023].

**Fig 2:**
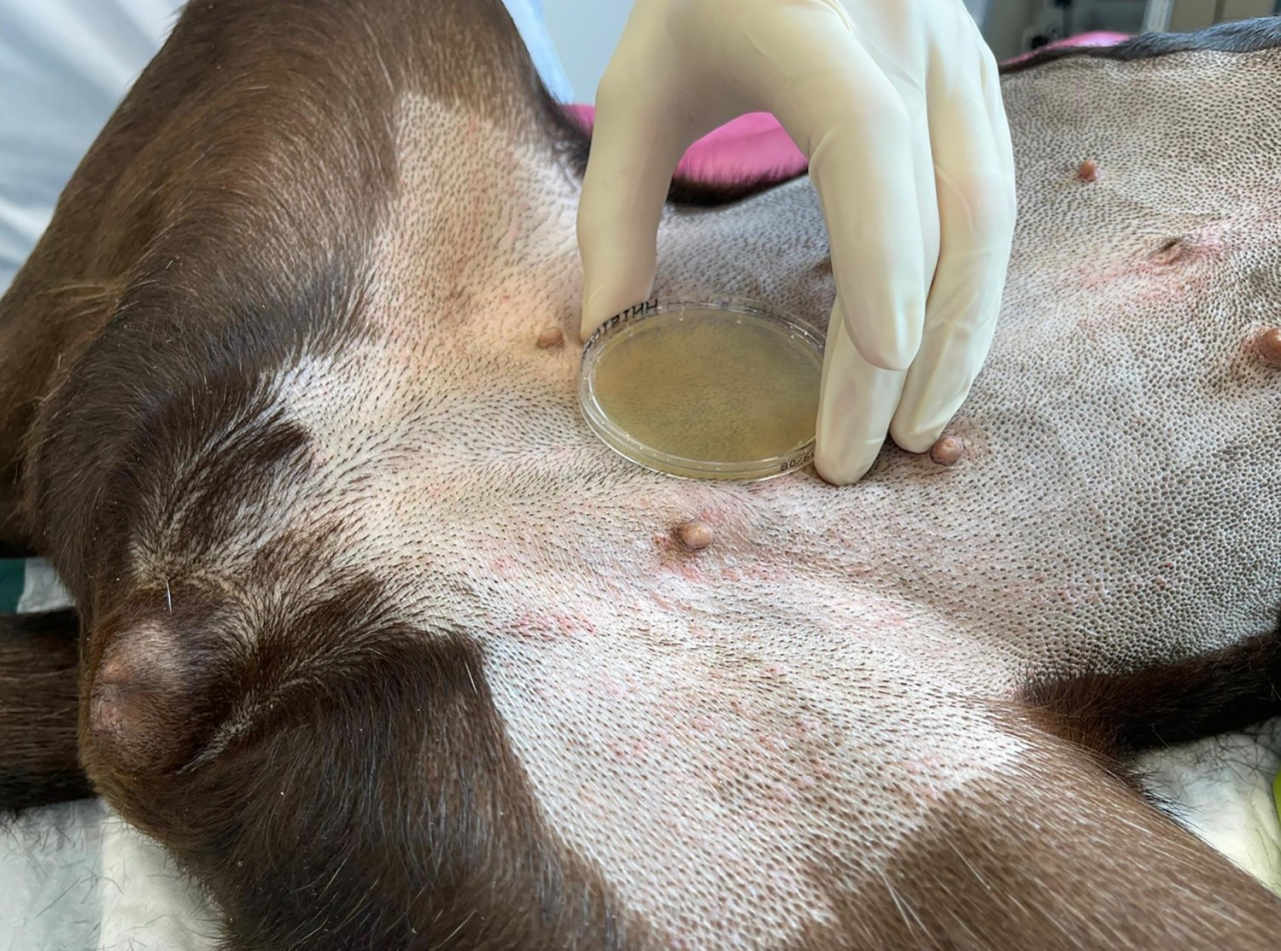
Contact plate (CP) sampling on the same dog as shown in Fig 1. Printed under a CC BY license, with permission from Fabian Eigner, original copyright [2023].

Samples were taken using two different methods: the wet-dry swab technique (WDS), see Fig 1, as well as sampling via contact plates (CP), Fig 2. The WDS technique was performed as follows: sterile metal templates were designed to delineate a defined area of 25cm^2^. The template was placed on the skin area to be sampled, and a moistened (sterile 0.9% saline solution) sterile cotton swab was gently rolled over the entire skin area in a meander pattern, followed by a dry sterile cotton swab. Both swabs were transferred into the same sterile tube with 2.5 ml of a mixture of sterile 0.9% saline-Pepton solution containing Tween 80 (desinhibitor). For CP sampling, a contact plate with desinhibitor (Tween 80) (Thermo Fisher Diagnostics AG) of 25 cm^2^ was gently pressed on the skin for 10 seconds.

### Patient enrollment

Dogs were randomly assigned to one of the groups via lottery, with the person performing the sample analysis and bacterial counts (SS, SC) being blinded to the treatment process. The lottery was performed by selecting one of sixty folded pieces of paper from a bag that contained 30 names of group O and 30 of group C. The person taking the samples (FE) was not able to be blinded to the treatment process, as the different solutions used were visually distinguishable. The sample size was chosen based on comparable studies and set to n = 30 per group [22–24,26–29]. After induction of anesthesia (non-standardized protocols adjusted to the clinical needs of each dog) the surgical field was clipped, and the first samples were taken using both techniques (T1^WDS^ and T1^CP^ in two separate skin areas located centrally along the planned incision site). The skin was washed using sterile wet four-by-four swabs using octenidine dihydrochloride containing soap (Octenisan®, Schülke & Mayr AG, Frauenfeld, Switzerland) in an inward to outward fashion with a contact time of one minute in group O. In group C, the same process was performed using a pH neutral soap without any active antiseptic ingredients (Allercalm®, Virbac Schweiz AG, Opfikon, Switzerland). After soap removal and drying of the skin using sterile four-by-four swabs, the second samples were taken (T2^WDS^ and T2^CP^) on random new locations in the aseptic field. Attention was paid not to overlap any sample area. The washed area was then covered with a sterile drape and the patient moved to the operating theatre, where the drape was removed by a scrub nurse wearing sterile gloves. The skin was disinfected using sterile four-by-four swabs soaked in octenidine dihydrochloride containing alcohol solution (Octeniderm®, Schülke & Mayr AG, Frauenfeld, Switzerland) in group O, and 74.1% ethanol in 10% 2-propanol (Softasept N®, BBraun Medical AG, Melsungen, Germany) in group C. After one minute contact time and a glove change, the procedure was repeated followed by another minute of contact time.This procedure has been developed according to the manufacturer’s protocol for humans, with an additional repetition added (as no veterinary specific recommendation exists) [58]. The repetitive washing and disinfection have been included because dogs skin is usually more dirty and more difficult to clean compared to human skin. After skin disinfection, the third samples were taken (T3^WDS^ and T3^CP^) from new locations in the aseptically prepared field.

**Fig 3:**
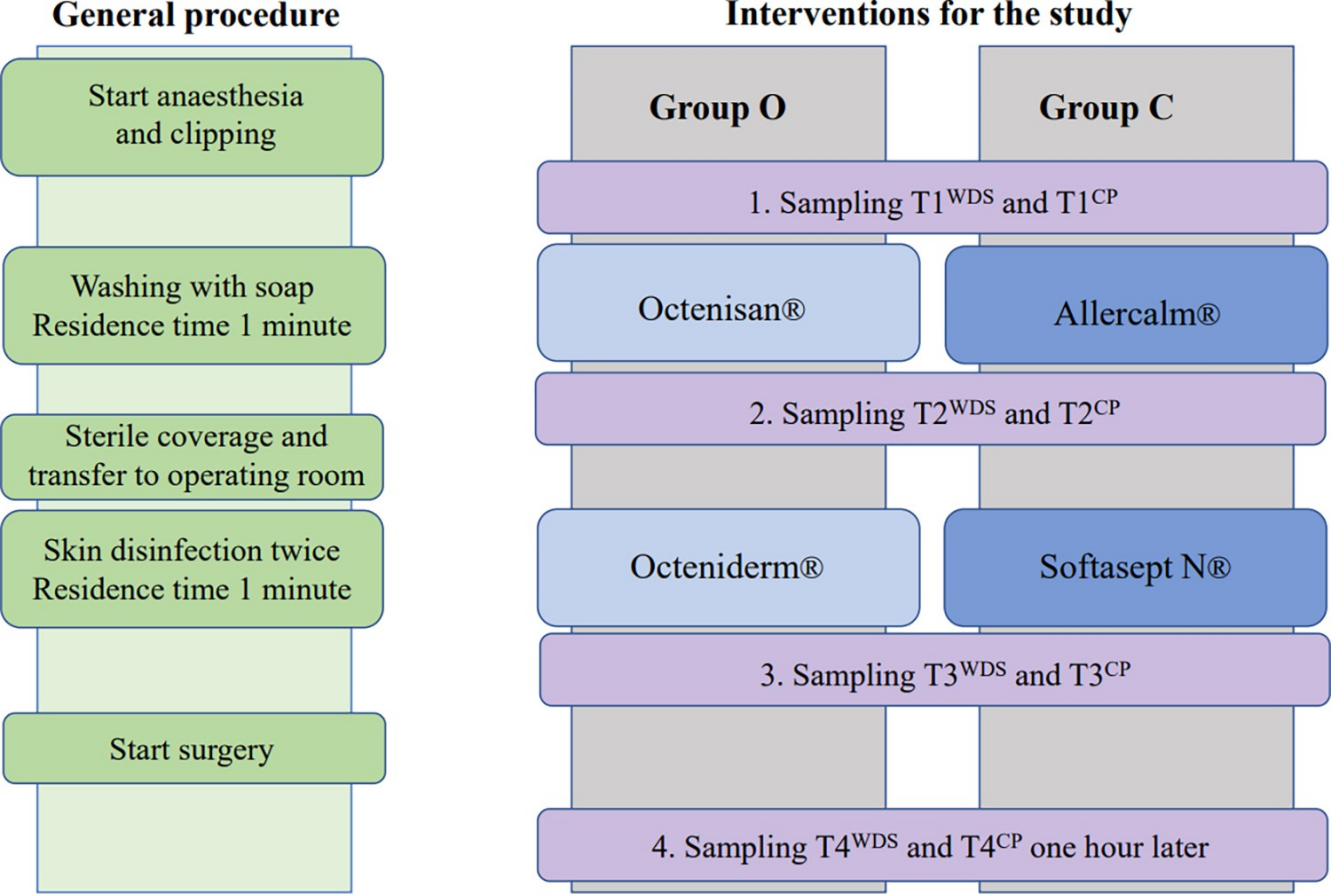
All the steps of skin preparation and sampling per group.

The patients were then draped for the surgery, and the procedure was started. The fourth sample (T4^WDS^ and T4^CP^) was taken under sterile conditions 60 minutes after T3 from a previously unsampled area in the sterile field. In cases where draping and surgery took less than 60 minutes, the patient was left anesthetized and draped till the last sample was completed. But this was the exception, since after T3 the surgeons needed time to drape and prepare the instruments, which is not considered within surgery time. The individual steps are depicted in Fig 3.

### Sample analysis

Directly after the procedure, the swabs and contact plates were transferred to the microbiology laboratory. CP were incubated under aerobic conditions at 37°C for 48 hours. Colony forming units per cm^2^ (CFU/cm^2^) were counted by a microbiologist who was blinded regarding the chosen antiseptic.

Both swabs and the 2,5 ml of a mixture of sterile 0.9% saline-Pepton solution containing Tween 80 (desinhibitor) were transferred to a small stomacher bag and homogenized for 60 s in 10 ml of peptone water in a stomacher. 50 μl of the suspension was plated with a spiral plater (Eddy Jet, IUL SA, Barcelona, Spain) onto TSA-agar (Thermo Fisher Diagnostics AG). The plates were incubated under aerobic conditions at 37°C for 48 hours. Colonies were counted by a colony counter and the result was calculated as CFU/cm^2^. The detection limit was <10 CFU/cm^2^.

All bacteria grown on TSA-Agar were then subcultured, and species identification was done using MALDI-TOF MS.

Groups were compared based on % reduction of CFU/cm^2^ over time from T1 to T2, T1 to T3 and T1 to T4.

### Statistics

The absolute number of CFU/cm^2^ was given in mean and range. Data summaries (scatter plots, histograms, means, medians, standard deviations, and ranges) were used to check the data for spurious observations and outliers and to check data distributions. All data were non-normally distributed based on Kolgomarov Smirnov testing, and comparisons were thus calculated using the Mann-Whitney U test. Statistical significance was set at P < 0.05. Equivalence testing was completed using a confidence interval of 90% and a Cohens’s d of 0.5.

## Results

A total of 61 dogs were enrolled, 30 were assigned to group O and 31 to group C. Details on patient specific data (breed, age, weight), surgical procedures (procedure type, anesthesia duration, surgery duration) and outcome are provided in S1 Table of the supporting information.

### Comparison of the sampling techniques

The comparison of bacteria detected by the two sampling techniques is displayed in Table 1. The number of detected bacteria was significantly different between CP and WDS technique at all timepoints. WDS detected significantly more bacteria in samples with high bioburden compared to CP sampling. However, the lowest number of CFU/cm^2^ detectable by WDS was “<10”, consequently it was not possible to adequately quantify low bioburdens, as seen after skin disinfection. Due to this important limitation, direct comparison of the antiseptic effect in both groups were done using CP results.

**Table 1:** Absolute numbers of bacteria detected in CFU/cm^2^ with CP vs. WDS sampling.

	CP		WDS		P
	Mean	STD	Mean	STD	
T1	8.18	4.8	632.64	2119.16	< 0.000
T2	1.44	3.04	663.87	4615.65	< 0.000
T3	0.01	0.06	10.49	2.84	< 0.000
T4	0.04	0.09	13.93	24.38	< 0.000

### Comparative performance of both skin preparation protocols

**Fig 4:**
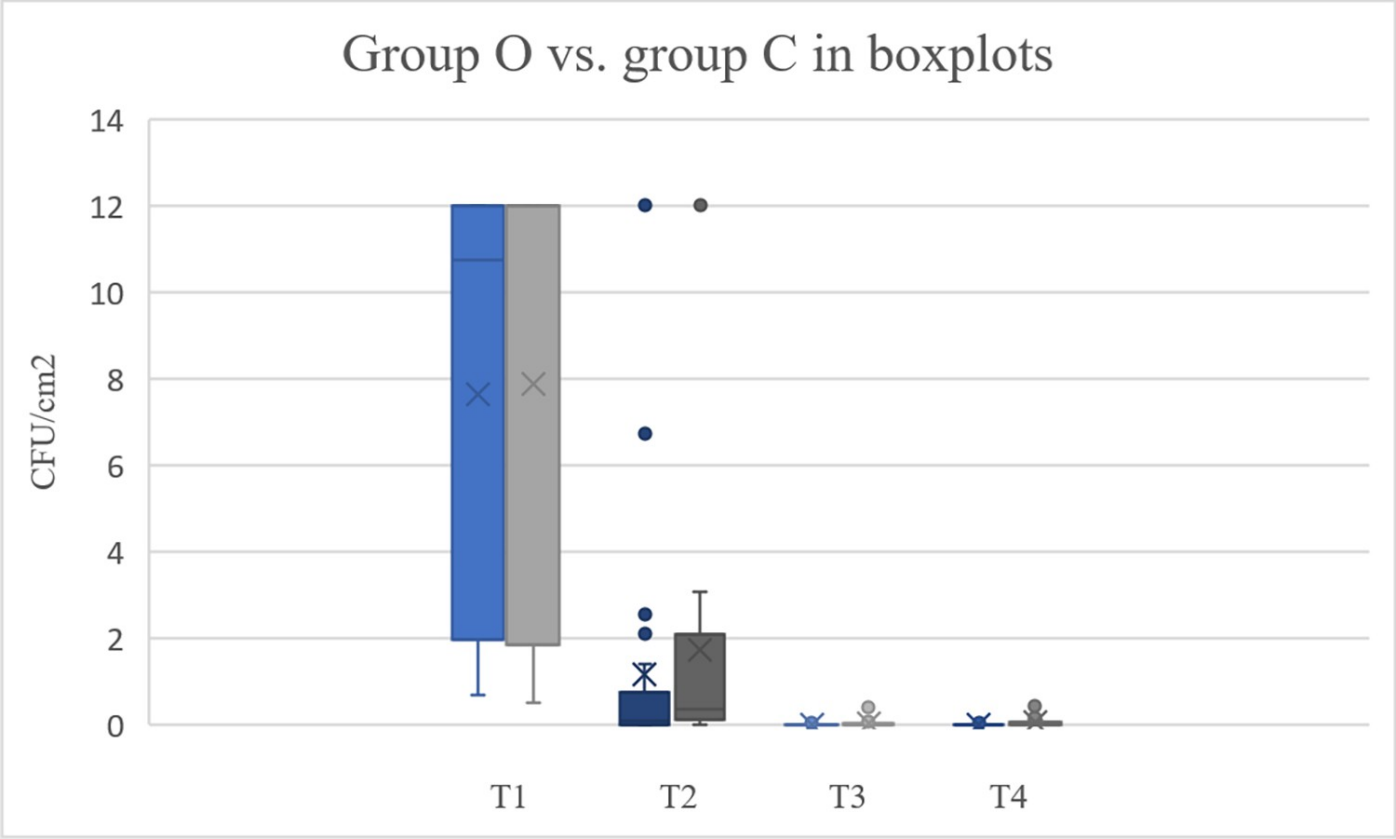
Box plot diagram of the bioburden given in CFU/cm^2^ for both groups (blue = group O, grey = group C).

The absolute detected numbers at all stages are visiblein Table 2. Despite small variations, the absolute number of bacteria before washing (T1) was comparable between groups. Washing with soap (T2) removed the majority of the bacteria in both groups. Disinfection further reduced skin bioburden at T3, but bacteria counts slowly increased within the next hour in both groups (T4). While no significant differences between groups were detected at T1 and T2, the absolute bacteria count was significantly lower in group O compared to group C at T3 and T4 (Fig 4). The relative reduction of bacteria given in percentage reduction between T1 and T2 was higher for group C, however this was not significant. Combination of washing and disinfection resulted in efficient skin decontamination in both groups, however group O significantly outperformed group C for both stages, meaning that in group O we detected significantly less bacteria in T3 and T4 compared to group C (Table 3).

**Table 2:** Absolute bacteria counts for both treatment groups given in CFU/cm^2^.

	*CFU/cm*^*2*^ *group O*		*CFU/cm2 group C*		*P*
	*Mean*	*STD*	*Mean*	*STD*	
*T1*	*7*.*8219*	*4*.*81087*	*8*.*4359*	*4*.*79610*	*0*.*456*
*T2*	*1*.*3665*	*3*.*12253*	*1*.*5738*	*3*.*04307*	*0*.*227*
*T3*	*0*.*0013*	*0*.*00718*	*0*.*0286*	*0*.*07990*	*0*.*019*
*T4*	*0*.*0116*	*0*.*03132*	*0*.*1062*	*0*.*22634*	*0*.*001*

**Table 3:** Relative reduction of the bacterial number over time.

*% bioburden reduction*	*group O*		*group C*		*P*
	*Mean*	*STD*	*Mean*	*STD*	
*reduction T1-T2*	*68*.*90*	*92*.*94*	*77*.*39*	*38*.*30*	*0*.*099*
*reduction T1-T3*	*99*.*99*	*0*.*06*	*99*.*70*	*0*.*75*	*0*.*018*
*reduction T1-T4*	*99*.*90*	*0*.*27*	*98*.*50*	*3*.*26*	*0*.*001*

### Testing for equivalence

Despite significant differences detected for both timepoints, the concrete difference was small. Prior to starting the study, a moderate effect (Cohen’s d > 0.05) was set for evaluation of clinical relevance (equivalence testing). Based on this prerequisite, the two treatment strategies are rated clinically equivalent for T1-T2 and T1-T3. At T1-T4 Cohen’s d was 0.64, therefore treatments cannot be considered equivalent any more after one hour.

### Isolated bacteria species

MALDI-TOF MS identified 55 different bacteria (Table 4) within the subcultures. The focus here was on the detected bacteria in T3 and T4, to see which bacteria were still present after disinfection. Nine bacterial species were identified in T3 and/or T4: *Staphylococcus epidermidis*, *Bacillus cereus*, *Staphylococcus capitis*, *Staphylococcus pseudintermedius*, *Micrococcus luteus*, *Staphylococcus cohnii*, *Staphylococcus warneri*, *Bacillus simplex* and *Staphylococcus haemolyticus*.

**Table 4:** Shows the cultured bacteria alphabetically per group and time, the bacteria in T3 and T4 are marked in grey, which were still detectable after disinfection.

	Group O	Group C	Group O	Group C	Group O	Group C	Group O	Group C
	T1	T1	T2	T2	T3	T3	T4	T4
*Acinetobacter lwoffii*	1	0	0	0	0	0	0	0
*Arthrobacter gandavensis*	1	0	0	0	0	0	0	0
*Bacillus cereus*	0	1	0	0	1	0	0	0
*Bacillus licheniformis*	0	2	0	0	0	0	0	0
*Bacillus megaterium*	0	3	1	0	0	0	0	0
*Bacillus mycoides*	1	2	0	0	0	0	0	0
*Bacillus niacini*	0	1	0	0	0	0	0	0
*Bacillus pumilus*	1	1	0	0	0	0	0	0
*Bacillus simplex*	0	1	0	0	0	0	0	2
*Bacillus subterraneus*	1	0	0	0	0	0	0	0
*Bacillus thuringiensis*	0	1	0	0	0	0	0	0
*Corynebacterium aurimucosum*	1	0	0	0	0	0	0	0
*Corynebacterium auriscanis*	1	0	0	0	0	0	0	0
*Corynebacterium mucifaciens*	0	0	1	0	0	0	0	0
*Corynebacterium tuberculostearicum*	0	1	0	0	0	0	0	0
*Curtobacterium flaccumfaciens*	0	0	1	0	0	0	0	0
*Dermacoccus* sp.	0	1	0	0	0	0	0	0
*Escherichia coli*	0	1	1	0	0	0	0	0
*Enterococcus hirae*	1	0	0	0	0	0	0	0
*Fictibacillus arsenicus*	1	0	0	0	0	0	0	0
*Kocuria carniphila*	1	0	0	0	0	0	0	0
*Kocuria kristinae*	1	0	0	0	0	0	0	0
*Macrococcus canis*	1	1	1	2	0	0	0	0
*Macrococcus caseolyticus*	1	0	0	0	0	0	0	0
*Macrococcus luteus*	1	0	0	0	0	0	0	0
*Malassezia pachydermatis*	1	0	0	0	0	0	0	0
*Microbacterium oleivorans*	1	0	0	0	0	0	0	0
*Micrococcus* sp.	2	1	0	0	0	0	0	0
*Micrococcus flavus*	0	0	1	0	0	0	0	0
*Micrococcus luteus*	2	7	1	3	1	3	4	5
*Moraxella canis*	1	1	0	0	0	0	0	0
*Paenibacillus amylolyticus*	1	0	0	0	0	0	0	0
*Paenibacillus provencensis*	0	1	0	0	0	0	0	0
*Psychrobacter sanguinis*	1	1	0	0	0	0	0	0
*Rothia endophytica*	1	0	0	0	0	0	0	0
*Rothia nasimurium*	1	0	0	0	0	0	0	0
*Serratia liquefaciens*	0	0	1	0	0	0	0	0
*Staph*. *(Staphylococcus) aureus*	0	1	0	0	0	0	0	0
*Staph*. *auricularis*	0	1	0	0	0	0	0	0
*Staph*. *capitis*	2	2	2	1	0	0	0	3
*Staph*. *cohnii*	0	1	0	0	0	0	0	1
*Staph*. *epidermidis*	4	8	0	0	0	0	1	1
*Staph*. *equorum*	0	0	0	1	0	0	0	0
*Staph*. *felis*	0	1	0	0	0	0	0	0
*Staph*. *haemolyticus*	0	0	0	0	1	0	0	0
*Staph*. *hominis*	1	4	0	1	0	0	0	0
*Staph*. *lugdunensis*	0	0	1	0	0	0	0	0
*Staph*. *petrasii*	1	0	0	0	0	0	0	0
*Staph*. *pettenkoferi*	1	0	0	0	0	0	0	0
*Staph*. *pseudintermedius*	4	5	4	7	0	0	0	1
*Staph*. *schleiferi*	1	0	0	0	0	0	0	0
*Staph*. *simulans*	1	0	0	0	0	0	0	0
*Staph*. *warneri*	1	2	1	0	1	1	1	1
*Streptococcus canis*	0	2	0	1	0	0	0	0
*Streptomyces violaceoruber*	1	0	0	0	0	0	0	0

### Complications

Two patients of group O and one patient of group C had a confirmed surgical site infection (SSI). This represents a SSI rate of 6.7% in group O and 3.3% in group C. Based on this small effect, our study does not have sufficient power to allow calculation of an statistical significant impact of the treatment on the SSI rate (power < 0.1, a future study would need a sample size of 798 dogs per group to reach a power of 0.8).

All three SSIs were deep and needed a wound revision surgery (Clavian Dindo grade III complications).

The first dog that developed an SSI was a pug after mastcell tumor removal in the inguinal region. Culture revealed *Streptococcus canis* at the time of the SSI as well as in T1, but not in T2, T3 nor T4.

The second SSI occurred in a French bulldog after cystotomy. The bacteria cultured from the SSI were *Staphylococcus pseudintermedius* and *Streptococcus canis*. The *Staphylococcus pseudintermedius* was phenotypically methicillin-susceptible.

On the same patient *Staphylococcus pseudintermedius* was also cultured from the skin in T1 and T2, but not in T3 nor T4.

The third SSI occurred in a Labrador Retriever after resection of an adenocarcinoma at the right shoulder area. The culture taken at the time of SSI identified *Staphylococcus pseudintermedius* and *Enterococcus faecium*, the *Staphylococcus pseudintermedius* was also phenotypically methicillin-susceptible.

Neither of the bacteria were cultured from the patient’s skin at any time before or during the procedure.

## Discussion

We were able to document a significant difference in the accuracy of detecting bacteria betweenWDS and CP sampling. While WDS identifies significantly more bacteria in situations with high bioburden, it is not useful to detect differences in cases with overall low bioburden, as the smallest possible number of bacteria to be detected is <10 CFU/cm^2^, and thus much higher than the residual load on the skin of dogs after disinfection using both protocols. Consequently, comparison of treatment groups was based on CP sampling.

To our knowledge there is currently no study that directly compares WDS sampling and CP sampling. The most established collection method on non-disinfected human skin is premoistened swabbing [59,60]. In contrast, most of the studies on dog’s skin were made with contact plates, especially for the evaluation of skin antisepsis [22–24,27,28,33]. The big advantage of the contact plate method is the increased sensitivity for detection of low numbers of the target organism [61]. Consistency in the same sampling technique is very important for comparative purposes, since for example with the swab technique heavier pressure picks up more microorganisms than light pressures [62]. For this reason, the same person (FE) took all samples in this study to decrease alterations.

As the dog skin is more dirty than human skin due to reduced daily hygiene, we routinely include a soap washing step in our protocol, as disinfection is not effective on grossly dirty skin. In both groups, washing with soap resulted in the highest reduction of CFU/cm^2^, 67 respectively 77% of the bacteria. That result is consistent with Kampf and Kramer (2004) who showed handwashing with regular soap without any active ingredients leads to a mean reduction of up to 2.4 log_10_ [63]. Of note, pH neutral soap performed equally well as octenidine containing detergent. Based on our results, we therefore recommend to use neutral skin friendly soap without additives to clean the skin, as the addition of a biocide does not seem to have any beneficial effect.

With respect to skin antisepsis, we had to decline our hypothesis, that the addition of a biocide would not result in an improved skin disinfection compared to a purely alcohol-based protocol. However, albeit being significant, the effects were small, both in absolute numbers (mean CFU/cm^2^ T3 group O 0.02 less than group C, mean CFU/cm^2^ T4 group O 0.09 less than group C) as well as with regard to % reduction (% reduction T1-T3 for group O 0.29% better than group C; % reduction T1-T4 for group O 1.4% better than group C).

Prior to the start of the study, we determined that the effect needs to be at least moderate, in order for it to be considered potentially relevant. When we use this assumption for equivalent testing, octenidine only outperforms alcohol at the latest time point.

This finding can be attributed to the so-called remanent effect that represents one of the reasons for adding a biocide in perioperative skin disinfection [47,64]. The significant difference in T4^CP^ confirms the residual effect of octenidine dihydrochloride in dogs, as described in humans [49,65,66]. This residual effect is caused by binding of octenidine to human epithelial cells and primary keratinocytes. Once bound, it cannot be removed easily but builds stable combinations with cell surfaces [38,47].

Our findings are comparable to the results of human medicine studies, even though the number of publications comparing the efficiency of octenidine dihydrochloride to other antiseptics is low [65–68]. As the addition of a biocide to the antiseptic protocol is not without potential risks, it is important to emphasize that equivalence testing only identified a clear benefit after 60 Minutes.

It has become apparent, that octenidine dihydrochloride can increase the antiseptic minimum inhibitory concentrations in *Staphylococcus epidermidis* and *Staphylococcus aureus* and that it induces a clinically decreased susceptibility to Penicillin in *Staphylococcus aureus* [3,69]. Cross-tolerance between octenidine dihydrochloride and gentamicin, colistin, amikacin, tobramycin, chlorhexidine and other biocides has also been described in *Pseudomonas aeruginosa* [70,71]. One suggested mechanism is mutation within the NorA or NorB efflux pumps [3,72].

In contrast to biocides like octenidine and chlorhexidine, tolerance against alcohol or acquired resistance mechanisms have been reported very rarely [73–75]. One of those studies showed strains of hospital acquired *Enterococcus faecium* after 2010 were 10-fold more tolerant to isopropanol than older isolates from 1997 [75]. In the context of clinical usage, alcohol tolerance in bacteria such as *Staphylococcus* spp. and *Streptococcus* spp. and cross resistance to antibiotics has not been reported yet [56].

For hand antisepsis alcohols without any additional biocides are clearly recommended [2,21]. But for the presurgical skin antisepsis of patients the combination of alcohols and longer lasting antimicrobial substances like chlorhexidine or octenidine is still recommended by the WHO [4]. Knowing the risk of resistances against biocides and cross resistances to antibiotics we should consider avoiding the use of additional biocides on top of alcohols for presurgical skin antisepsis in short surgeries, as it’s not clearly proven that additional biocides decrease the risk of SSI’s.

On a sidenote, this study was not designed to detect a potential impact of the two treatment protocols with respect to SSI prevention which represents a major limitation. Previous studies already failed to determine a direct correlation between a concrete bacterial load and the development of SSIs. [22,76–78]. The difficulty is that SSI is impacted by multiple factors. Due to the relatively low SSI rate in clean and clean contaminated procedures, studies that explicitly want to evaluate this correlation would need to standardize all other impacting factors in a large cohort of cases (around 800 per group based on our results) [8,79].

The SSI rate in our study was 6.7% (2/30) in group O and 3.3% (1/31) in group C, and is therefore comparable to rates previously described for clean and clean contaminated surgeries in veterinary medicine [8].

The isolated bacteria were identified with MALDI-TOF MS (matrix-assisted laser desorption ionization-time of flight mass spectrometry), because it’s fast, effective and relatively low in cost [80]. In total 55 different bacterial species were identified as seen in Table 4. Those bacteria are consistent with a healthy microbiome of dog’s skin [81,82]. Nine bacterial species were identified after disinfection or one hour later: *Bacillus cereus*, *Bacillus simplex*, *Micrococcus luteus*, *Staphylococcus capitis*, *Staphylococcus cohnii*, *Staphylococcus epidermidis*, *Staphylococcus haemolyticus*, *Staphylococcus pseudintermedius* and *Staphylococcus warneri*. We haven’t seen a pattern of specific bacteria per group. *Micrococcus luteus* was the most frequent isolate in T4 of both groups, but infections with this bacteria are very rare and usually affect immunosuppressive patients only [83].

In the three deep SSIs we were able to culture *Staphylococcus pseudintermedius* and *Streptococcus canis* twice and *Enterococcus faecium* once. *Staphylococcus pseudintermedius* has rapidly emerged as the most common cause of canine SSI worldwide, whereas *Enterococcus faecium* is another commensal of the dog’s intestines, which is occasionally found in SSIs. On the other hand, *Streptococcus* spp. are rarely seen in SSIs of dogs [1,8]. In all of those three cases of SSI, the bacteria found in the wound were not identified in T3 nor in T4. This concurs with the finding of another study, which could not see a significant correlation between pre- or postpreparation bacterial counts and SSI rate [78]. Interestingly, in two out of three SSI the same bacteria were isolated before disinfection. This could indicate a more profound role of the resident rather than the transient skin flora in the context of SSI development. Another theory could be that the bacteria were still present in the surgical site at T3 or T4, just not cultured. However with these few SSI numbers, this is purely speculative.

In conclusion, we were able to document a remanent effect of octenidine alcohol preparations for skin disinfection. Nevertheless, given the potential drawbacks and the overall excellent performance of both protocols, we recommend to reserve octenidine containing antiseptics for procedures with expected duration of more than 60 minutes to avoid overuse and potential impact on resistance and tolerance formation [21,28]. Future controlled studies are required to determine a potential impact on SSI development.

## Supporting information

S1 Table S1 Table shows all the patients that were enrolled in this study by group, breed, age, body weight, sex, surgery type, surgery time, anesthesia time and the SSI outcome. (DOCX)

S1 Dataset Dataset group O shows the raw data of group O for statistic. (XLSX)

S2 Dataset Dataset group C shows the raw data of group C for statistic. (XLSX)
