# Supplementary material for: Efficiency of octenidine dihydrochloride alcohol combination compared to ethanol based skin antiseptics for preoperative skin preparation in dogs
Source: PLoS One. 2023 Nov 7;18(11):e0293211. doi: 10.1371/journal.pone.0293211 (PMC10629653; doi:10.1371/journal.pone.0293211)
Supplement: S1 Table — (DOCX) [file pone.0293211.s001.docx]

*Supplementary table 1 shows all the patients that were enrolled in this study by group, breed, age, body weight, sex, surgery type, surgery time, anesthesia time and the SSI outcome*

| **group** | **Breed** | **Age y** | **Body weight kg** | **Sex** | **Surgery type** | **Surgery time in minutes** | **Anesthesia time in minutes** | **Outcome SSI pos/neg** |
| --- | --- | --- | --- | --- | --- | --- | --- | --- |
| O | Malinois | 5 | 21 | f/n | OHE (ovarihysterectomy) & mastectomy | 110 | 296 | neg |
|  | Golden Retriever | 2 | 27.5 | f | ectopic ureter | 165 | 245 | neg |
|  | Miniature Schnauzer | 11 | 6.8 | f/n | Splenectomy & Mucocele | 118 | 224 | neg |
|  | Mixed | 6 | 38 | m/n | Softtissue sarcoma removal forelimb | 75 | 195 | neg |
|  | Mixed | 1 | 28.8 | f/n | Mastcelltumor removal hindleg | 65 | 145 | neg |
|  | Alpenländische Dachsbracke | 1 | 13.4 | f/n | lap OE (laparoscopic ovariectomy) | 43 | 140 | neg |
|  | Jack Russell Terrier | 3 | 9.3 | f/n | Lymphadenectomy popliteal | 65 | 100 | neg |
|  | French Bulldog | 4 | 12.1 | m | Cystotomy | 76 | 159 | pos (deep SSI) |
|  | Golden Retriever | 5 | 34.5 | m | Mastcelltumor removal thoracic wall | 52 | 174 | neg |
|  | German Shepherd | 4 | 38 | f/n | Ovary rest syndrome | 95 | 147 | neg |
|  | Border Terrier | 10 | 9.5 | m/n | Thoracoscopic pericardectomy | 135 | 237 | neg |
|  | Golden Retriever | 10 | 33.4 | f/n | Digit amputation | 60 | 192 | neg |
|  | Weimaraner | 4 | 31 | f/n | Splenectomy | 62 | 140 | neg |
|  | Rough Collie | 10 | 17.8 | f/n | Splenectomy & OHE | 120 | 180 | neg |
|  | Mixed | 7 | 26 | f/n | OHE & mastectomy | 125 | 200 | neg |
|  | Golden Retriever | 8 | 41 | f/n | Mastcelltumor removal neck | 55 | 149 | neg |
|  | Border Collie | 10 | 24.4 | f/n | Mastcelltumor removal hind leg | 50 | 144 | neg |
|  | Cocker Spaniel | 13 | 11.2 | f | Nephrectomy | 68 | 155 | neg |
|  | Mixed | 9 | 19 | m/n | Cystotomie | 85 | 189 | neg |
|  | Lhasa Apso | 8 | 10.4 | f/n | Adrenectomy | 66 | 144 | neg |
|  | Mixed | 2 | 20.5 | f/n | Chronic diaphragmatic hernia | 105 | 160 | neg |
|  | Akita | 9 | 26 | f/n | Amputation forelimb Osteosarcoma | 121 | 206 | neg |
|  | Australian Cobberdog | 2 | 9.5 | f/n | lap OE | 25 | 94 | neg |
|  | Mixed | 8 | 16 | m | Mastcelltumor removal pectoral | 80 | 230 | neg |
|  | Jack Russell Terrier | 11 | 10 | m/n | Mastcelltumor removal neck | 67 | 172 | neg |
|  | French Bulldog | 10 | 8.5 | f/n | Softtissue sarcoma removal shoulder | 76 | 165 | neg |
|  | Mixed | 1 | 11 | f/n | Esophagus myotomy | 120 | 206 | neg |
|  | Pudelpointer | 7 | 4.6 | f/n | Ovary rest syndrome | 160 | 260 | neg |
|  | Labrador Retriever | 6 | 30 | m | Adenocarcinoma shoulder | 80 | 168 | pos (deep SSI) |
|  | Chihuahua | 7 | 4.8 | m | Cystotomy | 120 | 202 | neg |
| C | Mixed | 1 | 24.1 | f/n | lap OE | 38 | 114 | neg |
|  | Pug | 11 | 8.8 | m/n | Mastcelltumor removal inguinal | 115 | 210 | pos (deep SSI) |
|  | Jack Russell Terrier | 10 | 6.5 | f/n | OHE | 60 | 140 | neg |
|  | Mixed | 7 | 26.7 | f/n | Ovary rest syndrome | 115 | 210 | neg |
|  | Sibirian Husky | 1 | 21.3 | f/n | lap OE | 39 | 135 | neg |
|  | Gordon Setter | 11 | 32.5 | m | Splenectomy & Liverbiopsy | 100 | 161 | neg |
|  | French Bulldog | 0.5 | 9.2 | f/n | lap OE | 48 | 126 | neg |
|  | Labrador Retriever | 9 | 28.9 | f/n | Mastcelltumor removal hindleg | 110 | 225 | neg |
|  | Mixed | 1 | 18 | f/n | lap OE | 43 | 139 | neg |
|  | Dalmatian | 1 | 22.4 | f/n | lap OE | 35 | 92 | neg |
|  | Sibirian Husky | 1 | 20 | f/n | lap OE | 50 | 112 | neg |
|  | Australian Cobberdog | 2 | 9 | f/n | lap OE | 50 | 115 | neg |
|  | Boxer | 5 | 39.7 | m | Mastcelltumor removal shoulder | 77 | 193 | neg |
|  | Bernese Mountain Dog | 1 | 30 | f/n | lap OE | 39 | 123 | neg |
|  | Hungarian Pointer | 0.5 | 13.2 | f/n | lap OE | 30 | 109 | neg |
|  | French Bulldog | 1 | 12 | f/n | lap OE | 35 | 105 | neg |
|  | Mixed | 1 | 12 | f/n | lap OE | 32 | 106 | neg |
|  | Mixed | 10 | 30.4 | m/n | Lipoma removal axilla | 35 | 135 | neg |
|  | Lagotto Romagnolo | 1 | 12.8 | f/n | lap OE | 35 | 101 | neg |
|  | French Bulldog | 1 | 9 | f/n | OE | 40 | 95 | neg |
|  | Dogo Argentino | 1 | 34.5 | f/n | OE | 60 | 120 | neg |
|  | Mixed | 3 | 12 | f/n | OHE | 40 | 111 | neg |
|  | Labrador Retriever | 11 | 26.5 | m/n | Nephrectomy | 105 | 175 | neg |
|  | Mixed | 1 | 22.7 | f/n | lap OE | 43 | 110 | neg |
|  | Mixed | 1 | 16.7 | f/n | lap OE | 30 | 115 | neg |
|  | Labradoodle | 1 | 13.5 | f/n | lap OE | 30 | 124 | neg |
|  | Labrador Retriever | 1 | 29.9 | f/n | lap OE | 39 | 116 | neg |
|  | Jack Russell Terrier | 13 | 7.9 | f/n | Lipoma removal thoracic wall | 37 | 96 | neg |
|  | Hungarian Pointer | 1 | 22 | f/n | OHE | 75 | 150 | neg |
|  | Samoyed | 1 | 18 | f/n | lap OE | 65 | 126 | neg |
|  | Labrador Retriever | 1 | 25 | f/n | lap OE | 35 | 100 | neg |
